# Morphological, Phylogenetic and Physiological Studies of Pico-Cyanobacteria Isolated from the Halocline of a Saline Meromictic Lake, Lake Suigetsu, Japan

**DOI:** 10.1264/jsme2.ME11329

**Published:** 2011-12-27

**Authors:** Kaori Ohki, Kazumasa Yamada, Mitsunobu Kamiya, Shinya Yoshikawa

**Affiliations:** 1 Faculty of Marine Bioscience, Fukui Prefectural University, 1–1, Gakuencho, Obama, Fukui 917–0003, Japan

**Keywords:** cyanobacteria, isolation, Lake Suigetsu, meromictic lake, pico-cyanobacteria

## Abstract

Small cyanobacteria (<2 μm, pico-cyanobacteria) are abundant in waters deeper than the oxic-anoxic zone in the halocline of a saline meromictic lake, Lake Suigetsu, Fukui, Japan. We have isolated 101 strains that were grouped into six groups by means of the phycobiliprotein composition and sequence homology of the intergenic spacer between the 16S and 23S rRNA genes. Significant growth was observed under weak green light (1.5 μmol m^−2^ s^−1^, approx. 460 to 600 nm), whereas the cells died under white light at even moderate intensities. The isolates grew in a wide range of salinities (0.2 to 3.2%). Tolerance to sulfide varied: four groups grew in medium containing sulfide, however, two groups did not. None of the isolates were capable of anoxygenic photosynthetic (PS-II independent photosynthetic) growth using sulfide as an electron donor. All groups were included within fresh and brackish water of *Synechococcus/Cyanobium* clade, but they were not monophyletic in the 16S rRNA gene-based phylogenetic tree. The physiological properties of pico-cyanobacteria showed that they had the ability to survive in unique physicochemical environments in the halocline of this saline meromictic lake.

A saline meromictic lake is a permanent stratified water body that consists of three layers, a surface oxic mixolimnion, a bottom anoxic monolimnion and a compact transition layer (halocline) between the mixolimnion and monolimnion. The halocline is characterized by a steep salinity gradient and the presence of an oxic-anoxic boundary zone where the oxygen concentration is zero and hydrogen sulfide begins to be detected. In many cases, a dense microbial community is present around the oxic-anoxic boundary zone, and a unique microbial loop is created (1, 4, 6, 14, 19, 39).

Lake Suigetsu, a member of the Mikata Lake Group (“Mikata Goko”, Japanese for “consists of five lakes”), is located in the central part of the coast of Wakasa Bay along the Sea of Japan in Fukui Prefecture, Japan. At a maximum depth of 34 m, it is the second deepest lake in the Mikata Lake Group. Lake Suigetsu has the typical characteristics of a saline meromictic lake (18); fresh water flows into Lake Suigetsu from a neighboring freshwater lake, Lake Mikata, and drains into a brackish lake, Lake Kugushi, through the artificial Urami channel that was constructed in the middle of the 17^th^ century. Because Lake Kugushi opens into Wakasa Bay through a narrow and shallow river (Hayase River), seawater enters Lake Suigetsu periodically via Lake Kugushi during high tide. As a result, stratification with a surface freshwater layer and deep saline water layer occurs in Lake Suigetsu. The large difference in specific density between the surface and deeper water prevents vertical mixing where the two layers are separated by a steep halocline (0.2 to 1.4%) at a depth of about 2 to 15 m. Dissolved oxygen in the deep saline water is consumed by the decomposition of organic substances (anoxic) where a permanent oxic-anoxic boundary zone is formed near the middle of the halocline (at a depth of 4 to 6 m) (20–22, 30). Sulfate derived from seawater is reduced to sulfide by sulfate-reducing bacteria deeper than the oxic-anoxic boundary zone (15, 16). Only very weak light (less than 10 μmol m^−2^ s^−1^ even at noon on a clear summer day, unpublished) penetrates the oxic-anoxic boundary zone. Light of longer wavelength (>600 nm) decays with increasing depth, with shorter wavelength light (400 to 600 nm) dominating near the bottom of the halocline (30).

The major primary producers in the anoxic layer in the stratified lake are anoxygenic phototrophs, namely photosynthetic bacteria, if light is available. In the halocline of Lake Suigetsu, the abundance of bacteriochlorophyll *e*-containing sulfur bacteria, *Chlorobium* species, has been reported using pigment analysis (30, 38), PCR-denaturating gel electrophoresis of the 16S rRNA gene (17) and a clone library constructed by PCR products of the α-subunit of the reverse dissimilatory sulfite reductase (*dsrA*) gene (25) using the particle fractions of lake waters.

In the summer of 2005, we found that very small unicellular cyanobacteria (pico-cyanobacteria <0.5–1×1–2 μm) were abundant (from 1 to 5×10^5^ cells mL^−1^) not only in the oxic mixolimnion but also deeper than the oxic-anoxic boundary zone in the halocline (30). In July to early September 2005, the maximum cell density of pico-cyanobacteria was observed 1 to 2 m deeper than the oxic-anoxic boundary zone but not in the mixolimnion (30). Later, pico-cyanobacteria were found to be distributed in the halocline throughout the year, although seasonal fluctuations of cell densities were observed.

The distribution of *Synechococcus* or *Synechococcus*-like pico-cyanobacteria in the anoxic layer of stratified lakes has been reported by epifluorescence microscopic (6) and epifluorescence and electron microscopic (4) observations and *in situ* 16S rRNA gene analysis (14). These reports together with our findings mentioned above indicate that pico-cyanobacteria, oxic-phototrophs, inhabit the anoxic environment of stratified lakes; however, cells of pico-cyanobacteria have few morphological characteristics that allow their identification. There is little phylogenetic information available concerning the physiological properties of pico-cyanobacteria found in the anoxic layer of stratified lakes.

The question arises regarding which physiological properties of these pico-cyanobacteria enable them to survive in the unique physicochemical environment of Lake Suigetsu, and whether these Lake Suigetsu pico-cyanobacteria are from a clade within a broader radiation of pico-cyanobacteria. To answer these questions, pico-cyanobacteria were isolated from the halocline of Lake Suigetsu and studied for their morphological, phylogenetic and physiological properties.

## Materials and Methods

### Isolation of pico-cyanobacteria

Primary samples for the isolation of pico-cyanobacteria were collected from deeper than the oxic-anoxic boundary zone in the halocline in the central part of Lake Suigetsu (35°35′N, 135°52′E) in July 2005, and July and August 2006. Water samples were mixed with an equal volume of culture media with 0.8, 1.6 or 3.2% salinity containing agarose (1.5% w/v, A0169; Sigma-Aldrich Inc., St. Louis, MO, USA, called agarose medium hereafter), and poured into Petri dishes. The medium contained salts (25 g NaCl, 0.3 g CaCl_2_, 0.7 g KCl and 9 g MgSO_4_ L^−1^) and was diluted with distilled H_2_O to obtain different salinities, and then nutrients were added as follows: 0.5 g NaNO_3_, 0.05 g K_2_HPO, 1 mL trace metal solution and 1 mL vitamin solution L^−1^(9). The pH of the medium was adjusted using Tris-HCl buffer (0.2 g L^−1^; pH 8.0). A single colony on agarose was picked up and inoculated into about 5 mL liquid medium (omitting agarose from agarose medium, called liquid medium hereafter). After cells in liquid medium became visible, a small aliquot was removed, mixed with agarose medium, and poured into Petri dishes. The procedures, colony picking up into liquid medium and plating, were repeated three or more times. Isolation was performed under dim light conditions (daylight-type fluorescent lamps covered with semi-opaque plastic plates, 3 to 5 μmol m^−2^ s^−1^) at 20°C. As all isolates grew well in salinity of between 0.4 to 1.6%, they were maintained in liquid medium at salinity of 0.8%.

### Pigment analysis

Absorption spectra of intact cells were measured using a spectrophotometer equipped with a head-on type photomultiplier (U3300; Hitachi, Tokyo, Japan). Absorption spectra of isolated phycobilisomes (PBS) were measured with a spectrophotometer equipped with a side-on type photomultiplier (UVmini-1240; Shimadzu Co., Kyoto, Japan). The methods for isolation of PBS and the estimation of phycobiliprotein composition were the same as those used in the previous paper (27).

### Morphological observations

Morphological observations were performed using an epifluorescence microscope (BX51; Olympus, Tokyo, Japan). The filter sets used for autofluorescence emitted from pico-cyanobacteria (excitation with green light and monitored at red wavelengths), autofluorescence emitted from photosynthetic bacteria (excitation with blue light and monitored at IR wavelengths) and 4′6-diamidino-2-phenylindole stained DNA (excitation with UV light and monitored at blue wavelengths) were DM570/BP530-550/BA575IF/DM570, DM570/BP530-550/41035IR/DM570 and M400/BP330-385/BA420/DM400, respectively. Fluorescence invisible wavelength was photographed using a CCD camera DP70 (Olympus). Because photosynthetic bacteria emitted autofluorescence at IR wavelengths, they were photographed using a CCD camera ORCA-C7780 (Hamamatsu Photonics, Hamamatsu, Japan) that is sensitive to both visible and IR lights. Ultrastructure of the pico-cyanobacteria cells was observed using an electron microscope (JEM1210; JEOL, Tokyo, Japan). Specimens were prepared for transmission electron microscopy as described previously (29).

### Phylogenetic analysis

Extraction methods for genomic DNA were the same as those of Porter (33). The primer sets for PCR amplified most of the 16S rRNA gene and the intergenic spacer (ITS) between the 16S and 23S rRNA genes. The amplification conditions for PCR were the same as those used in our previous report (28). The partial 16S rRNA gene sequences from 58 operational taxonomic units (OTUs) were aligned using the ClustalW algorithm as implemented in BioEdit 7.0.9 (10), and 1,396 bp alignment data were used for molecular phylogenetic analyses. Gaps were treated as missing data in the analysis, and ‘*Synechococcus elongatus*’ PCC6301 was used as an out-group. Maximum likelihood analysis was performed using PAUP*4.0b10 (37) based on the GTR+I+G model selected by the Akaike Information Criterion in the Modeltest 3.7 (34). Bootstrap analysis for ML was performed based on 100 replications of a heuristic search with a nearest neighbor interchange algorithm generated from re-sampled data. For Bayesian inference analysis, the GTR+I+G model was selected using MrModeltest 2.3 (26). Markov chain Monte Carlo iterations were conducted for five million generations sampling every 100 generations using MrBayes 3.1.2 (36).

The sequences of the 16S rRNA gene and the ITS between the 16S and 23S rRNA genes of the isolates were deposited in DDBJ under accession numbers AB610885 to AB610896.

### Physiological properties

The growth of isolates at different light intensities and salinities was assessed by inoculating Erlenmeyer flasks containing 80 mL liquid medium. Cells in the exponential growth phase were inoculated into fresh medium at a cell density of *ca.* 10^6^ cells mL^−1^ and incubated under various light intensities. To obtain different light intensities, fluorescent lamps were covered with semi-opaque plastic plates and layers of mesh cloth. To obtain green light (460 to 600 nm), fluorescent lamps were covered with a green plastic filter (No. 2092; Rohm and Hass, Philadelphia, PA, USA).

To determine the growth rate under different salinities, cells in the exponential growth phase cultured in medium at 0.8% salinity were collected and inoculated into the media at 0.2, 0.4, 0.8, 1.6 or 3.2% at a cell concentration of *ca.* 10^6^ mL^−1^. The light intensity was 8 and 15 μmol m^−2^ s^−1^ for phycoerythrin (PE)-containing and PE-free strains, respectively.

To evaluate the growth under various experimental conditions, an aliquot of cell suspension from each culture was collected every 24 h and stored at 4°C until counting after fixing with glutaraldehyde (1% v/v). The fixed cells were collected onto a polycarbonate membrane filter (0.2 μm pore size; Adovantec K020A047; Toyo Roshi Kaisha, Tokyo, Japan), and the number of autofluorescent cells was directly counted under an epifluorescent microscope. The specific growth rate, μ (h^−1^), was determined from the growth rate in the exponential growth phase using three independent experiments with duplicate cultures and expressed as the mean±SD. Statistical significance was judged by the t-test.

Growth in the presence of sulfide was performed by inoculating the cells into sulfide-containing medium prepared as follows: medium was buffered with 2-(4(2-hydroxyethyl)-1piperazinyl) ethanesulfonic acid-NaOH buffer (0.1 g L^−1^, pH 7.3) instead of Tris to keep the pH at around 7.3. About 30% of the added sulfide becomes hydrogen sulfide at this pH (12). After autoclaving and cooling down, sterilized N_2_ gas was bubbled for 1 min, and then filter-sterilized NaHCO_3_ solution (0.2 g L^−1^) and the appropriate amount of saturated Na_2_S solution were added to the medium. The pH was adjusted with 1 M H_2_SO_4_. The pH of the medium was kept at around 7.3 throughout the experiment period. Cells in the exponential growth phase in sulfide-free medium were inoculated into sulfide-containing medium at a cell concentration of ~10^6^ mL^−1^. Cells inoculated into sulfide-free medium were used as a control. Glass serum bottles (30 mL) were fully filled with medium and cells, and then capped with a rubber stopper and sealed with an aluminum cap. Serum bottles were incubated for 7 d under light intensity of 3 μmol m^−2^ s^−1^. Sulfide concentration was determined using the same methods as those used in our previous work (30). Inhibition of growth in the presence of sulfide was estimated from the ratio of the cell number increase in sulfide-containing medium to sulfide-free medium during 7 d incubation, and the sulfide concentration showing 50% inhibition was determined. In some experiments, 3-(3,4-dichlorophenyl)-1,1-dimethylurea (DCMU, 2×10^−5^ M) was added 24 h after inoculation. Survival in the presence of sulfide determined whether cells re-started growth in sulfide-free medium after 7 d incubation in sulfide-containing medium. The data are the mean±SD of three separate cell counts from triplicate cultures.

The fluorescence kinetics was measured at room temperature with an F-2000 fluorescence spectrophotometer (Hitachi, Tokyo, Japan) with a 435 nm excitation light (full width at half maximum, 10 nm) and monitored at 685 nm emission light (full width at half maximum, 5 nm). Cells incubated for 7 d in sulfide-containing medium were harvested by centrifugation, washed twice with fresh medium without sulfide, and re-suspended in the same medium. The steady state fluorescence level, F_s_, was recorded 1 min after excitation, and then DCMU (10^−5^ M) was added. The maximum fluorescence level, F_max_, was recorded 1 min after addition of DCMU. All measurements were performed after the cells had been pre-incubated in the dark for 30 min.

## Results

### Morphology

**Fig. 1:**
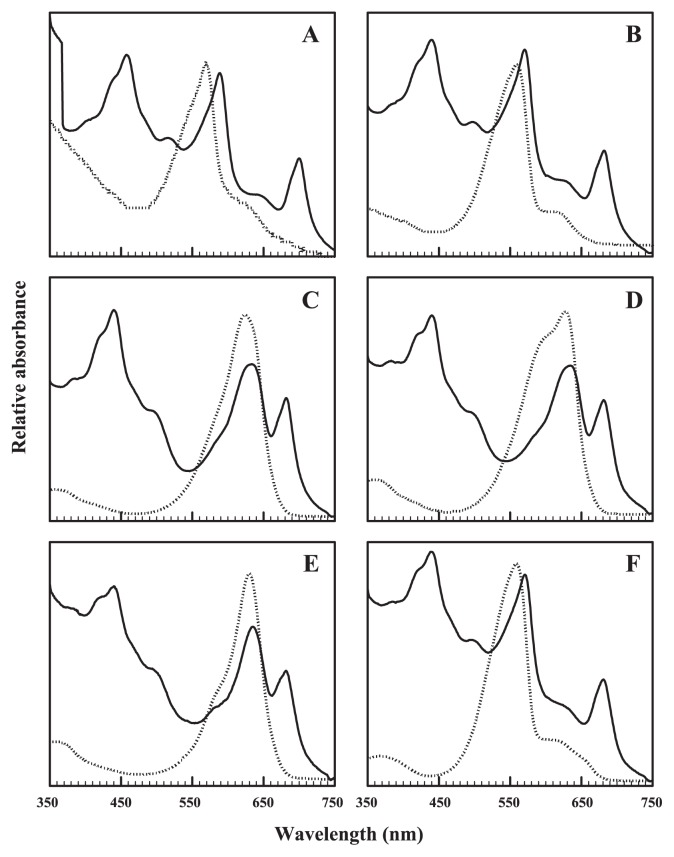
Absorption spectra of intact cells (solid line) and isolated phycobilisomes (dashed line) from pico-cyanobacteria. (A) Group I (CR2), (B) group II (CR3), (C) group III (CG3), (D) group IV (CG4), (E) group V (CG2), (F) group VI (CR5).

Both photosynthetic bacteria and pico-cyanobacteria were abundant in deeper than the oxic-anoxic boundary zone within the halocline of Lake Suigetsu (Fig. S1). Pico-cyanobacteria colored pink or green were recovered from water samples deeper than the oxic-anoxic boundary zone where O_2_ was not detected at the time of sampling (Table S1). Some physicochemical properties of the water layers from where pico-cyanobacteria were recovered are summarized in Table S1. Sulfide was detected at concentrations between 70 to 2,100 μM and the salinity was 1.0 to 1.3%. One hundred and one strains were obtained and grouped into six by the color of the cells (Table 1) and the sequence similarity of the DNA sequence of the intergenic spacer (ITS) between the 16S and 23S rRNA genes (Table 2, lower half of the matrix). The cells of groups I, II and VI were pink whereas those of groups III, IV and V were green (Fig. S2). Absorption spectra of intact cells and isolated phycobilisomes (PBS, Fig. 1) showed that the major photosynthetic pigments were phycoerythrobilin-containing phycoerythrin (PE) and C-type phycocyanin (PC) for groups I, II and VI (Fig. 1 A, B and F) and PC for groups III, IV and V (Fig. 1, C, D and E). None of the isolates had phycourobilin-containing PE or phycoerythrocyanin. The molar ratios of phycobiliprotein (PBP) revealed that groups I, II and VI had PBS with a large amount of PE, while group III, IV and V had PE-free PBS (Table 1). The sequence of ITS between 16S and 23S rRNA genes of the representative strains within the same group (group I; CR1 vs. CR2, group II; CR3 vs. CR4, group III; CG1 vs. CG3) was almost identical (≥99.8%, Table 2). Groups II and III had almost identical ITS sequences (99.9%, Table 2); however, the PBP compositions of groups II and III were different (PE-containing for group II and PE-free for group III, Fig. 1 B vs. C). The sequence similarities among groups II and III, I, IV, V and IV were less than 84.1% and 97.6% identity for ITS between 16S and 23S rDNA genes (Table 2, lower half of the matrix) and the 16S rDNA gene (Table 2, upper half of the matrix), respectively.

**Fig. 2:**
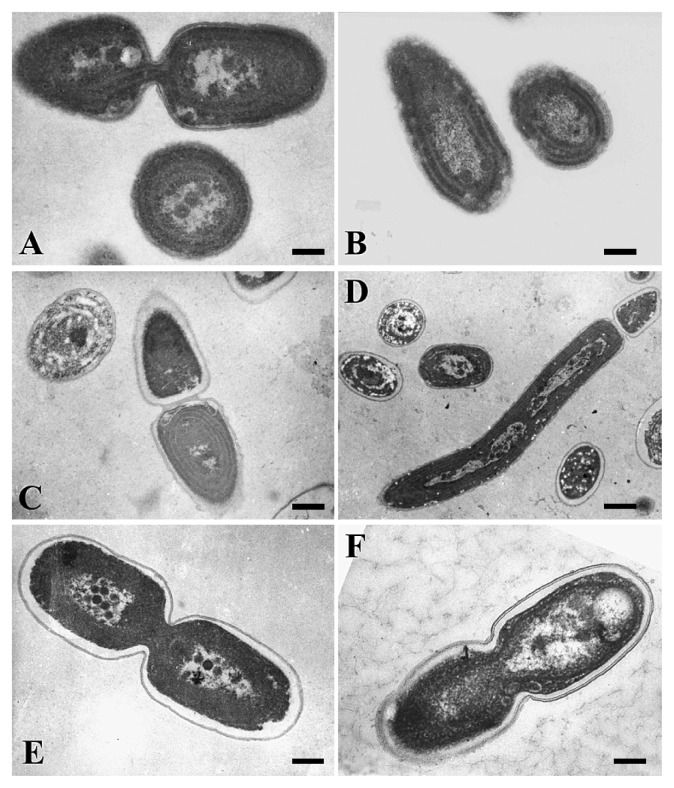
Transmission electron micrographs of pico-cyanobacteria isolated from the halocline of Lake Suigetsu. (A) Group I (CR2), (B) group II (CR3), (C) group III (CG3), (D) group IV (CG4), (E) group V (CG2), (F) group VI (CR5). Scale bars, 200 nm for (A), (B), (C), (E), (F) and 500 nm for (D).

Cell sizes of all isolates were very small at 0.5 to 1 μm in diameter and width for spherical and rod-shape cells, respectively. Cell division formed two daughter cells of equal size in groups I (CR2), II (CR3), III (CG1), V (CG2) and VI (CR5) (Fig. 2A, B, C, E and F; Fig. S3A, B, C, E and F). The cells of group IV (CG4) often became long (up to 15 μm in length). Unequal cell division of such long cells produced daughter cells of various sizes (Figs. 2D and S3D). Staining DNA with 4′6-diamidino-2-phenylindole revealed that multiple DNA replications occurred prior to cell division in the long cells of group IV (CG4, Fig. S4A vs. B). No isolates formed a well-defined sheath layer, whereas the strains of group V (CG2) excreted mucus substances into medium. Thylakoids of all isolates were peripheral and oriented parallel to the cytoplasmic membrane (Fig. 2). Several carboxysomes were observed in the central part of the cells.

### Phylogenetic relationships

**Fig. 3:**
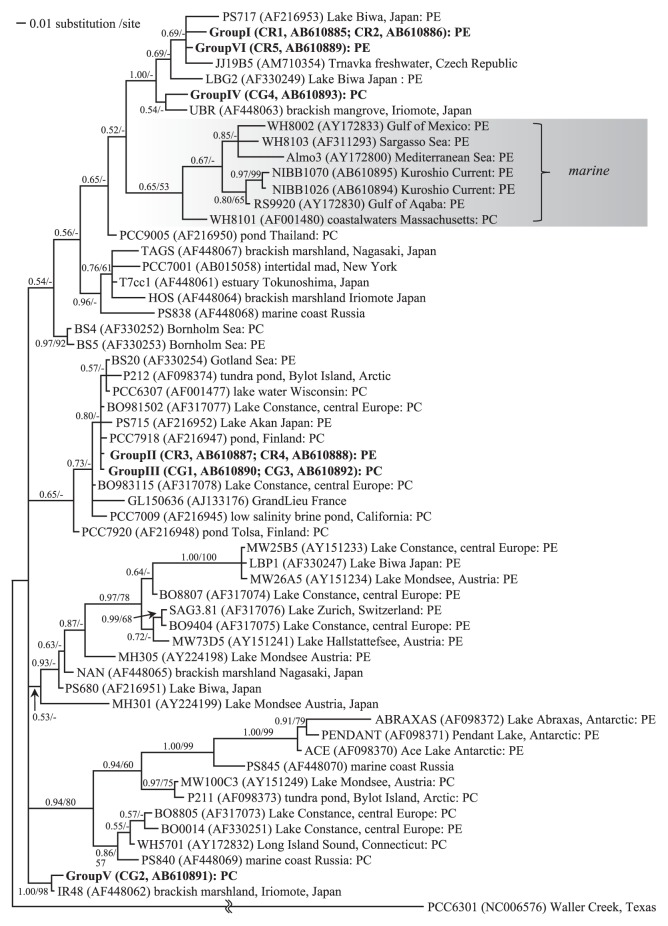
Bayesian phylogenetic tree inferred from the 16S rRNA gene sequences. The corresponding posterior probabilities (left) and bootstrap values from ML (>50%; right) are given on each branch. Pigment information (PE, phycoerythrin rich; PC, phycocyanin rich) is based on Crosbie *et al.*(5). Accession numbers are shown in parentheses.

Nine strains from six groups, all of which were included within the ‘*Synechococcus*’/‘*Cyanobium*’ clade, were divided into three clusters (Fig. 3). Group II (CR3, CR4) and III (CG1, CG3) were closely related to strains isolated from freshwater environments, *e.g.*, Lake Akan, Japan (PS715) and Lake Constance, central Europe (BO98152). Group V (CG2) formed a clade with the sample from brackish marshland on Iriomote Island, Japan (IR48) with high bootstrap support. Although Group I (CR1, CR2), Group IV (CG4) and Group VI (CR5) were included in the same clade that consisted of fresh or brackish water samples, the relationship within this clade was unresolved.

### Physiological properties

**Fig. 4:**
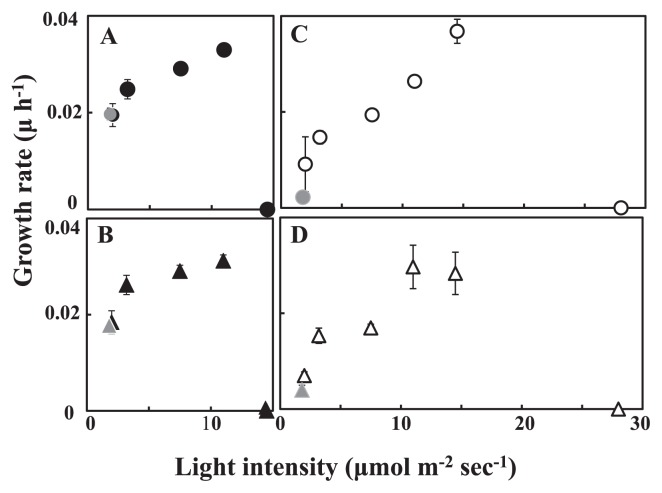
Specific growth rate of pico-cyanobacteria grown under different light intensities. Phycoerythrin-containing groups: (A) group I (CR2) grown under white light (black circles) or green light (gray circles); (B) group II (CR3) grown under white light (black triangles) or green light (gray triangles). Phycoerythrin-free groups; (C) group III (CG3) grown under white light (white circles) or green light (gray circles); (D) group IV (CG4) grown under white light (white triangles) or green light (gray triangles). Cells were cultured under white light of various intensities or under green light (460 to 600 nm) at 1.5 μmol m^−2^ s^−1^. Specific growth rate, μ (h^−1^), was determined from the increase in cell numbers during the exponential phase. Results are the mean±SD of three independent experiments with duplicate cultures. Error bar was omitted when±SD was too small to be shown.

Two PE-containing groups, groups I and II, and two PE-free groups, groups III and IV, were cultured under various light intensities to know whether the isolates were able to utilize the weak light penetrating the halocline. Strains CR2, CR3, CG3 and CG4 were used as representatives of groups I, II, III and IV, respectively. All groups showed significant growth even under weak white light as low as 2 μmol m^−2^ s^−1^ (Fig. 4). The maximum growth rate of PE-containing groups (I, II, Fig. 4A and B) was slightly but significantly higher than that of PE-free groups (III, IV, Fig. 4C and D) under light intensities lower than 11 μmol m^−2^ s^−1^ (*n*=12, *P* <0.001). Growth was maximal around light intensities of 11 and 15 μmol m^−2^ s^−1^ for PE-containing and PE-free groups, respectively. Cells were bleached irreversibly under white light at relatively high intensities (>15 μmol m^−2^ s^−1^ for PE-containing groups and >25 μmol m^−2^ s^−1^ for PE-free groups). As the light quality around the bottom layer of the halocline of Lake Suigetsu was predominantly green (<580 nm, 30), growth under green light was also examined. Both PE-containing and PE-free-groups grew slowly but significantly under weak green light at 1.5 μmol m^−2^ s^−1^.

**Fig. 5:**
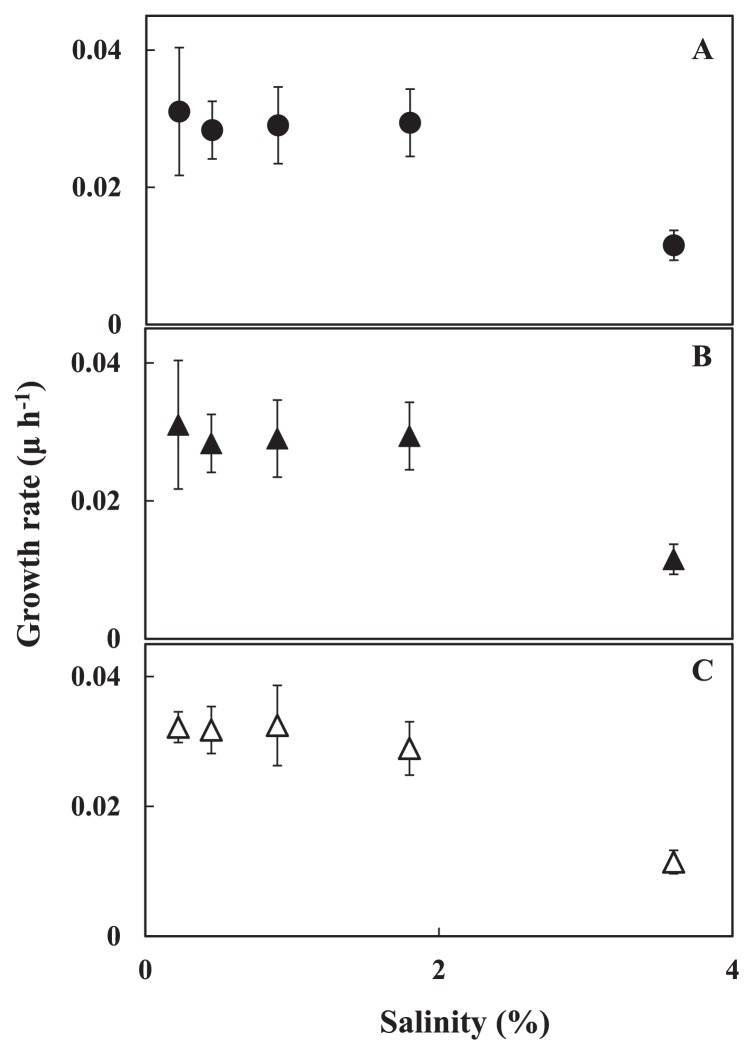
Specific growth rate of pico-cyanobacteria grown in medium at different salinities. Group I (CR2, A), group II (CR3, B), group IV (CG4, C). Light intensity was 8 μmol m^−2^ s^−1^ (groups I and II) or 15 μmol m^−2^ s^−1^ (group IV). Specific growth rate, μ (h^−1^), was determined from the increase of cell numbers during the exponential growth phase. Results are the mean±SD of three independent experiments with duplicate cultures.

Halocline environments are also characterized by a change in salinity. Three strains isolated from different depths (group II, CR3, 8 m; group III, CG3, 9 m; group IV, CG4, 10 m) were cultured in media of various salinities. To simulate the salinity changes that occur in natural environments, the concentrations of the salts in the medium were changed but those of the nutrients were kept constant (see, Material and Methods), and the maximum growth rate of pico-cyanobacteria in media of different salinities was determined (Fig. 5). Although the cells were pre-cultured in medium with 0.8% salinity, they began to grow without a distinctive lag after transferring to media of different salinities (data not shown). The maximum growth rate occurred at 0.2 to 1.6% salinity where in this range the differences were insignificant (*n*=6, *P* >0.01). Significant inhibition was observed at 3.2% salinity (*n*=6, *P* <0.05).

**Fig. 6:**
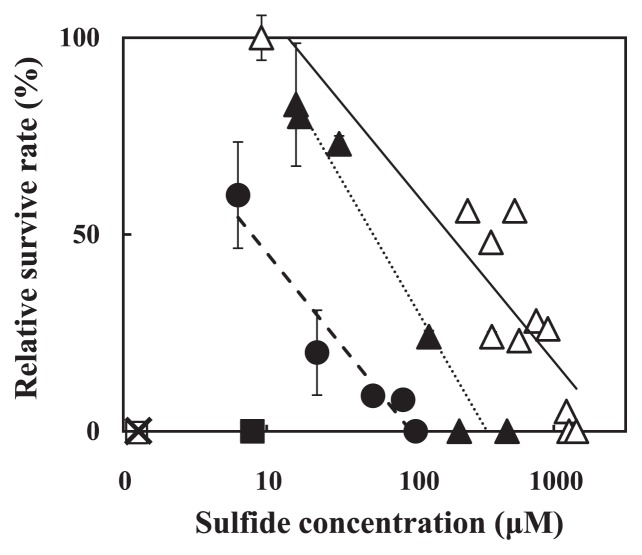
Effects of sulfide on the growth of pico-cyanobacteriaano-bacteria. Group I (CR2, black circles), group II (CR3, black triangles), group III (CG3, black triangles), group IV (CG4, white triangles, group VI, (CR5, crosses). Linear regression lines for Group I, group III and group IV are shown by dashed lines (r^2^=0.97), dotted lines (r^2^=0.96) and straight lines (r^2^=0.82). The sulfide concentration in the medium was determined after 7 d incubation. The relative inhibition of growth was estimated using the ratio of cell number increase in sulfide-containing medium compared to sulfide-free medium during 7 d incubation. Results are the mean±SD of three separate counts from triplicate cultures. Error bar was omitted when±SD was too small to be shown.

Another prominent physicochemical factor found in the halocline of Lake Suigetsu is the presence of sulfide. To determine whether pico-cyanobacteria have tolerance to sulfide, cell growth was examined in a medium containing sulfide. The relative growth rate in the presence of various concentrations of sulfide compared to the control without sulfide was determined during 7 d incubation. Because added sulfide decreased abiotically (*e.g.* leaking) by about 100 μM during 7 d incubation, the sulfide concentration in Fig. 6 was the value measured at the end of each experiment. In this experiment, clones CR2, CR3, CG3, CG4, CG2 and CR5 were used as representatives of groups I, II, III, IV, V and VI, respectively. Group IV showed the highest tolerance to sulfide; significant growth was observed in the presence of sulfide as high as 1,000 μM. In contrast, tolerance to sulfide of groups I and II was relatively low; complete inhibition of cell proliferation was observed by sulfide at about 110 to 220 μM (concentrations at the end of the experiment); however, groups I and II survived 7 d incubation in medium with sulfide higher than 500 μM; growth began again when cells were washed and transferred to sulfide-free medium (data not shown). Sulfide concentrations (at the end of experiments) showing 50% inhibition of growth in groups I, II and IV were 3.8 μM (r^2^=0.97), 50 μM (r^2^=0.96) and 180 μM (r^2^=0.82), respectively. Group V also showed tolerance to sulfide; however, statistical data could not obtained because the cells adhere to each other with a mucus substance excreted from cells. Groups III and VI did not grow in medium containing hydrogen sulfide (<100 μM, concentration at the beginning of the experiments). None of the isolates grew in sulfide-containing medium when 3-(3,4-dichlorophenyl)-1,1-dimethylurea (DCMU, 10^−5^ M) was added. Steady-state chlorophyll *a* fluorescence (F_s_) increased to the maximum fluorescence level (F_max_) when DCMU (2×10^−6^ M) was added to cell suspensions of groups I (CR2), II (CR3) and IV (CG4) incubated in the presence of sulfide for 7 d, although the value of F_max_ F_s_^−1^ was reduced to about one-third of the control cells. The F_max_ level of the cells incubated with sulfide for 7 d was not decreased by the addition of sulfide (data not shown).

## Discussion

Morphological features, the small size, reproduction by transverse binary fission in a single plane, peripheral thylakoids and lack of a structured sheath (Fig. 2, S3) indicate that our isolates are Form genus IV (‘*Cyanobium*’) or XIII (‘*Synecochoccus*’) of the Subsection I cyanobacteria (11). This identification is supported by the molecular phylogenetic data. The six pico-cyanobacteria groups were divided into three subclades and all of these clades belonged to the fresh and brackish water ‘*Synechococcus*’/‘*Cyanobium*’ clade (Fig. 3).

Our physiological studies show how pico-cyanobacteria acclimate to the unique physicochemical environments found in the halocline in Lake Suigetsu. They have the ability to utilize weak light for growth (Fig. 4). Our isolates in groups I (CR2), II (CR3), III (CG3) and IV (CG4) were able to grow under weak light, whereas cells were bleached irreversibly and died under white light at even moderate intensities. The efficiency to use weak light was significantly higher in phycoerythrin (PE)-containing groups, groups I (CR2) and II (CR3), than in PE-free groups, groups III (CG3) and IV (CG4) (*n*=12, *P* <0.001 for both white and green light). Phycobilisomes (PBS) with large quantities of PE (Table 1, Fig. 1 A and B) were able to utilize weak light more efficiently than PE-free PBS (13).

Pico-cyanobacteria isolates have tolerance to salinity changes (Fig. 5). The difference in salinity was about 1.0% between the upper and lower layers of the halocline of Lake Suigetsu (30). Further, the salinity fluctuated widely, the highest being 1.2% and the lowest 0.13% around the oxic-anoxic boundary zone in the halocline during 2007 to 2009 (unpublished data). Our isolates showed similar growth constants in salinity between 0.2 to 1.6% (*n*=6, *P* >0.01). Abrupt growth after transferring to different salinities was also observed. The data suggest that pico-cyanobacteria have developed the ability to acclimate to salinity changes, although the detailed mechanisms are unknown.

In addition, some pico-cyanobacteria isolates tolerate sulfide (hydrogen sulfide) (Fig. 6). It is known that hydrogen sulfide is a potent inhibitor of oxygenic photosynthesis where it is thought to poison the oxidation site of the PSII by interacting with Mn-stabilized protein (24, 32). Tolerance to sulfide among cyanobacteria differs among species (4, 7, 8, 23, 31, 32, 35). Further, the filamentous cyanobacterium, ‘*Oscillatoria limnetica*’, has the ability to grow utilizing hydrogen sulfide as an electron donor to perform anoxygenic photosynthesis in which only PSI is involved (2, 3).

The ability of sulfide-dependent anoxygenic photosynthesis in cyanobacteria has often been evaluated in short-term (< several hours) with CO_2_ assimilation in the presence of sulfideand3-(3,4-dichlorophenyl)-1,1-dimethylurea(DCMU); however, the ability for short-term anoxygenic CO_2_ assimilation does not always correlate with anoxygenic growth. For example, ‘*Aphanothece halophytica*’ (‘*Cyanothece*’ sp. PCC7418) and ‘*Prochrolothrix hollandica*’ Sag 10.89 were able to assimilate CO_2_ for several hours anoxigenically using sulfide as an electron donor; however, they did not grow under the same conditions (7, 35). Therefore, we evaluated sulfide tolerance using cellular proliferation during 7 d incubation in the presence of sulfide. Tolerance to hydrogen sulfide among our isolates was not the same. The high tolerance of group IV (CG4) to sulfide may reflect the environment where the strain was collected (hydrogen sulfide, 2100 μM, Table S1, 30). None of our isolates were capable of anoxygenic photosynthestic growth since growth in the presence of hydrogen sulfide was completely inhibited by DCMU. Results suggest that PSII of some pico-cyanobacteria is tolerant to hydrogen sulfide, because PSII-dependent fluorescence (DCMU-dependent increase of fluorescence, F_max_) was observed even after 7 d incubation with sulfide. The presence of PSII-dependent fluorescence (F_max_) in particle fractions containing pico-cyanobacteria collected from the halocline of Lake Suigetsu (30) supports this conjecture.

Our phylogenetic analysis showed that the six pico-cyanobacteria groups isolated from Lake Suigetsu are divided into three clusters that are evolutionary distant from each other. According to Crosbie *et al.*(5), non-marine pico-cyanobacteria were clustered into at least six to seven groups. Our isolates were closely related to the two groups of Crosbie *et al.*; groups I, IV and V were closely related to group E (Lake Biwa strain) and groups II, III and V were within group A (‘*Cyanobium gracilie*’ cluster). The relationship among pico-cyanobacteria groups was not supported by cell morphology, PBS composition, or the degree of hydrogen sulfide tolerance. For example, group II (CR3) (PE-containing and low sulfide-tolerant) and group III (CG3) (PE-free and not sulfide-tolerant) are closely related in the phylogenetic tree.

A question remains whether the isolated strain reflects the *in situ* pico-cyanobacterial population, because many phytoplankton are not cultivable; however, this concern may be alleviated, at least in part, because most of the pico-cyanobacteria found in the halocline of Lake Suigetsu were closely related phylogenetically to the isolated strain by the partial sequence of the PCR-amplified 16S rRNA gene using DNA retrieved from lake waters (Yoshikawa, personal communication). Since isolation and culture under artificial conditions necessarily has a certain bias, the results obtained in laboratory culture cannot be expanded directly to the physiological properties of pico-cyanobacteria in the natural habitat; however, tolerance to salinity changes and hydrogen sulfide and the ability to use weak, limited light for photosynthesis suggest that some pico-cyanobacteria found in the halocline contribute to biogeochemical cycling in Lake Suigetsu.

## Supplementary material



## Figures and Tables

**Table 1:** Data on the isolated pico-cyanobacteria used in this study

group	Number of strains	Collection		Strains used in this study	PBS-structurea)	
	
		date (mo/day/year)	Depth (m)		PE/APC (mol/mol)	PC/APC (mol/mol)
I	18	7/6/2005	7	Suigetsu-CR1	4.6	2.4
			8	Suigetsu-CR2		
II	49	7/6/2005	8	Suigetsu-CR3	4.1	2.1
			9	Suigetsu-CR4		
III	10	7/6/2005	7	Suigetsu-CG1	0	2.9
			9	Suigetsu-CG3		
IV	17	7/6/2005	10	Suigetsu-CG4	0	3.2
V	5	7/19/2006	8	Suigetsu-CG2	0	3.2
VI	2	8/14/2006	8	Suigetsu-CR5	6.9	1.8

a) Phycobilisome (PBS) structure expressed by the molar ratios of phycoerytyrin (PE) and phycocyanin (PC) to allophycocyanin (APC).

**Table 2:** Sequence similarities among the pico-cyanobacteria isolated from the halocline of Lake Suigetsu

Strains		I		II		III		IV	V	VI
			
		CR1	CR2	CR3	CR4	CG1	CG3	CG4	CG2	CR5
I	CR1	—	99.9	52.0	52.0	52.0	52.0	65.8	60.2	84.1
	CR2	100.0	—	52.0	52.0	52.0	52.0	65.8	60.2	84.1
II	CR3	97.2	97.2	—	100.0	99.9	99.9	59.2	58.5	52.0
	CR4	97.2	97.2	100.0	—	99.9	99.9	59.2	58.5	52.0
III	CG1	97.1	97.1	99.9	99.9	—	99.8	58.9	57.9	51.4
	CG3	97.1	97.1	99.9	99.9	100.0	—	58.9	57.9	51.4
IV	CG4	98.9	98.9	96.9	96.9	96.8	96.8	—	64.7	61.8
V	CG2	96.8	96.8	97.5	97.5	97.4	97.4	96.6	—	61.5
Vl	CR5	99.4	99.4	97.6	97.6	97.5	97.5	99.0	97.3	—

16S rRNA gene (upper half of the matrix) and the intergenic spacer between the 16S and 23S rRNA genes (lower half of the matrix) among the groups I to VI (Table 1).
