# Supplementary material for: Morphological, Phylogenetic and Physiological Studies of Pico-Cyanobacteria Isolated from the Halocline of a Saline Meromictic Lake, Lake Suigetsu, Japan
Source: Microbes Environ. 2011 Dec 27;27(2):171–8. doi: 10.1264/jsme2.ME11329 (PMC4036012; doi:10.1264/jsme2.ME11329)
Supplement: Supplementary file 1 [file 27_171_s1.pdf]

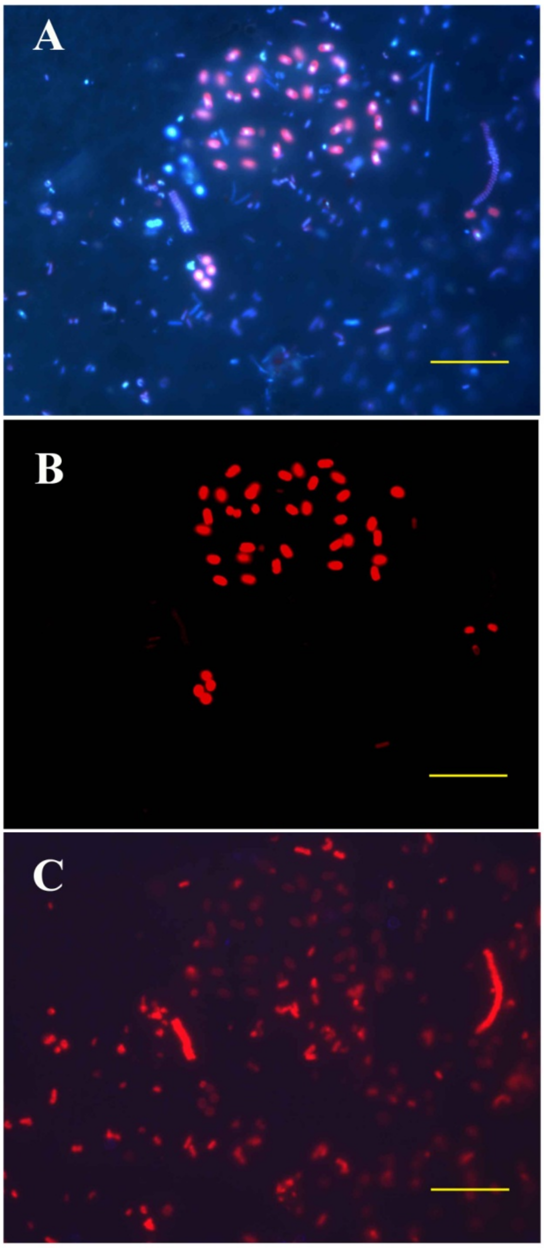

Fig. S1 Epifluorescence microscopic images of the particle fractions obtained from the halocline of Lake Suigetsu. (A) Images excited by UV-light and monitored at blue-wavelengths, (B) images excited with green-light and monitored in the orange to red-wavelengths, (C) images excited with green-light and monitored in IR wavelengths. Water samples were collected from a depth of 7 m (deeper than the oxic-anoxic boundary zone), and fixed with glutaraldehyde (1%). After the particle fractions were collected onto a polycarbonate membrane filter (0.2 µm; Adovantec K020A047, Toyo Roshi Kaisha Ltd, Tokyo, Japan), they were stained with 4'6-diamidino-2-phenylindole and photographed using an epifluorescence microscope (Olympus Co., Tokyo, Japan) equipped with a CCD camera ORCA-C7780 (Hamamatsu Photonics K.K., Hamamatsu, Japan) that is sensitive to both visible and IR lights. Scale bar, 5 µm.

Ohki et al. Fig. S1

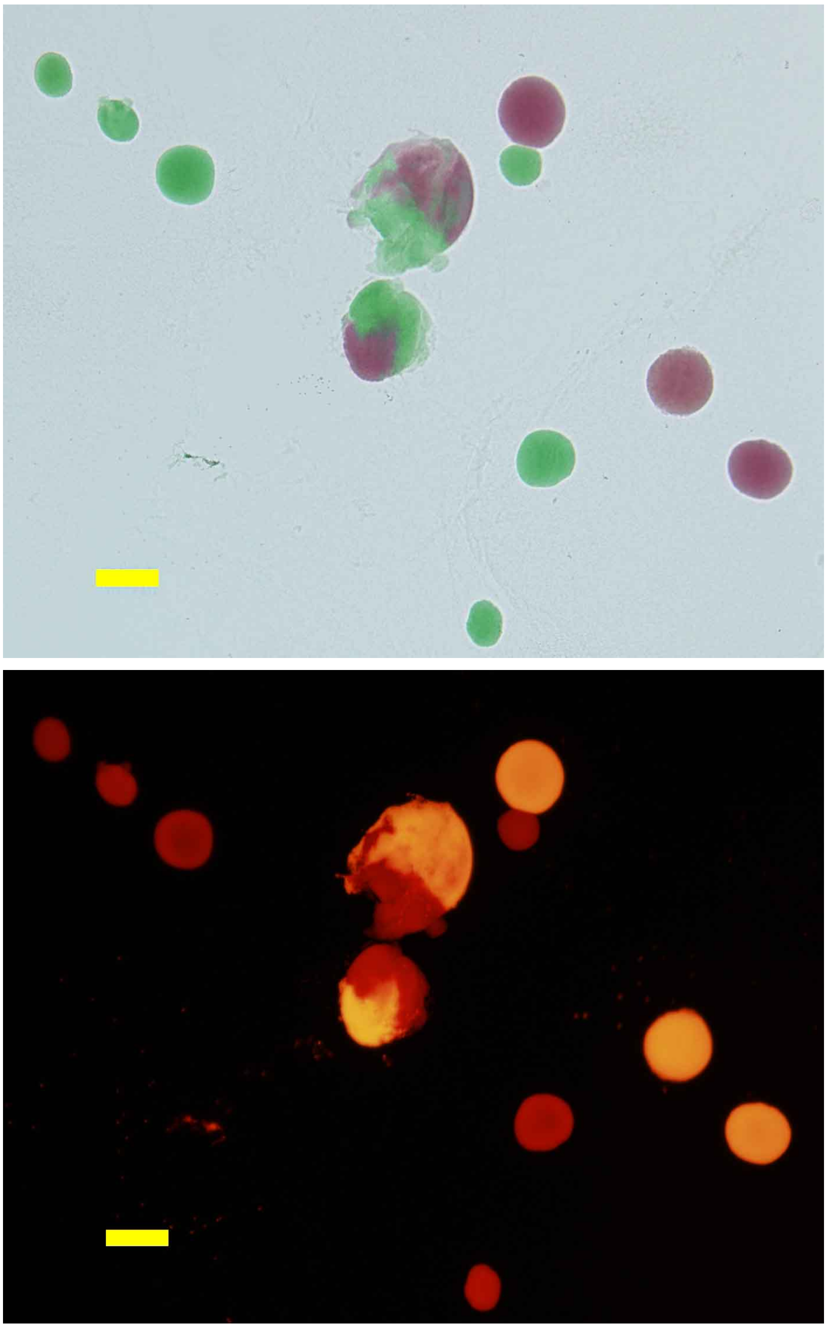

Fig. S2 Colony of pico-cyanobacteria recovered on an agarose plate. (A) light microscopic image, (B) epifluorescence microscopic image (excitation: green light, montered: orange to red-wavelengths). Scale bar, 50 μm.

Fig. S2, Ohki et al.

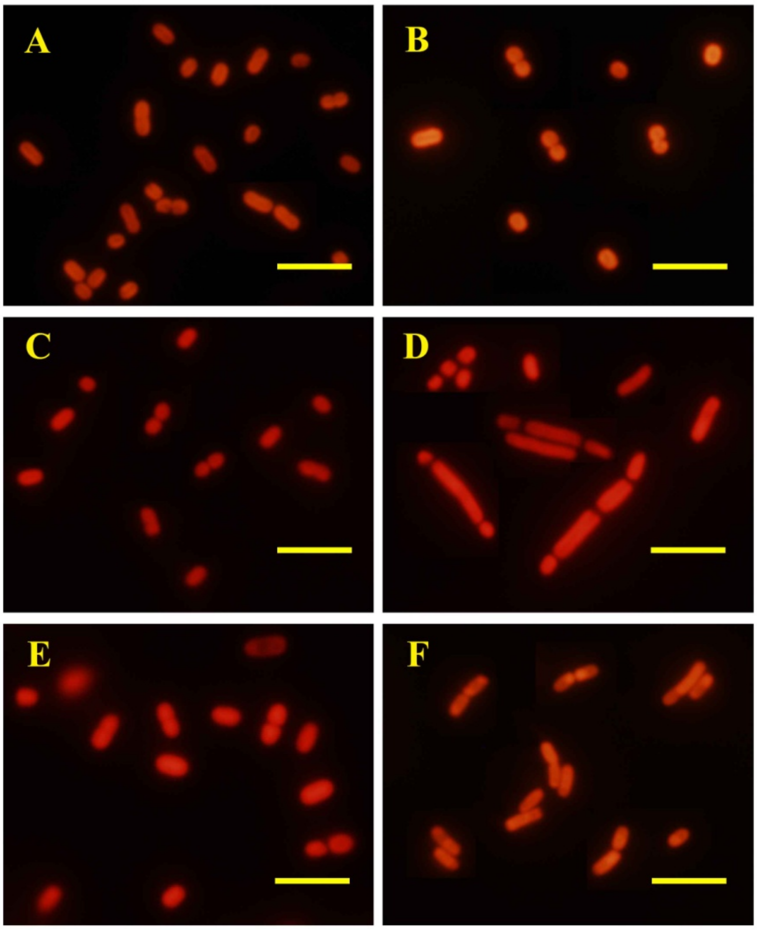

Fig. S3 Epifluorescence microscopic images of isolated pico-cyanobacteria (A) group I (CR2), (B) group II (CR3), (C) group III (CG3), (D) group IV (CG4), (E) group V (CG2), (F) group VI (CR5). Cells were fixed with glutaraldehyde (1% v/v) and collected onto a polycarbonate membrane filter (0.2 µm). Excitation light was green-light, and autofluorescence was monitored in the orange to red-wavelenghths. Scale bar, 5 µm.

Fig. S3, Ohki et al.

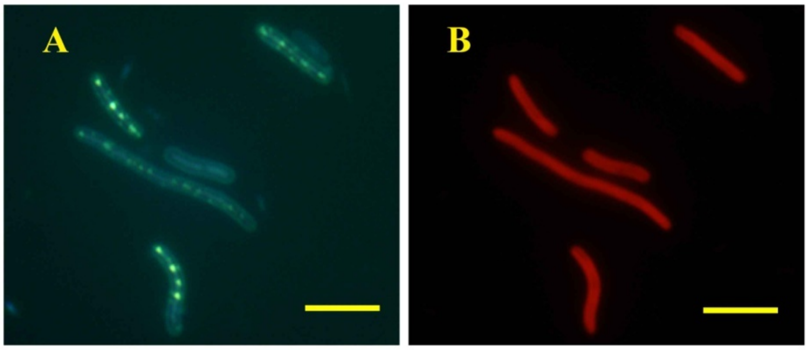

Fig. S4 Epifluorescence microscopic images of group IV (CG4). Cells were fixed with glutaraldehyde (1% v/v) and collected onto a polycarbonate membrane filter (0.2 µm) and stained with 4'6-diamidino-2-phenylindole. (A) image excited by UV-light and detected in the blue wavelengths, (B) image excited by green-light and monitored in the orange to red-wavelengths. Scale bar, 5 µm.

Fig.S4, Ohki et al.

Table S1. Some physicochemical properties of the water layers of Lake Suigetsu where pico-cyanobacteria were obtained.

Date	Depth (m)	Temper-ature (°C)	Salinity (pus)	O_2 (mg·L^{-1})	H_2S (μM)	Relative light intensities in visible wavelength (% of surface intensities)							
						398nm	447nm	488nm	542nm	589nm	629nm	678nm	707nm
7/6/2005[(1)]	7	20.3	10.8	0	69	1.12	0.26	0.05	0.07	0.03	0	0	0
	8	18.4	11.8	0	73	1.14	0.26	0.05	0.06	0.03	0	0	0
	9	16.7	12.9	0	1,405	1.15	0.27	0.05	0.07	0.02	0	0	0
	10	15.2	13.0	0	2,113	1.18	0.27	0.05	0.07	0.02	0	0	0
7/19/2006[(2)]	8	18.1	10.9	0	90	not determined							
8/14/2006[(2)]	8	17.9	11.3	0	360	not determined							

(1) Okada,M., Taniuchi,Y., Murakami,A., Takaichi,S., Yoshikawa,S., Ohki,K. Limnology 8. 271-280 (2007)

(2) Kondo & Okamoto, with permission.
